# A nursing intervention based on the Zentangle® method: Experiences of patients diagnosed with borderline personality disorder

**DOI:** 10.1016/j.ijnss.2024.03.004

**Published:** 2024-03-11

**Authors:** Ana Morales-Alonso, Ángela Iglesias-de-la-Iglesia, Miriam Alonso-Maza

**Affiliations:** a Dr. Rodríguez Lafora Hospital (Hospital Dr. Rodríguez Lafora), Madrid, Spain; b Department of Nursing and Stomatology, University Rey Juan Carlos, Alcorcón, Madrid, Spain

**Keywords:** Art therapy, Borderline personality disorder, Mental health, Nursing care, Zentangle

## Abstract

**Objective:**

The application of the Zentangle® Method in relation to relaxation and well-being has not been tested in patients with borderline personality disorder (BPD). This study was to analyze the practising Zentangle® experience in patients with BPD.

**Methods:**

With a phenomenological interpretative approach, this qualitative study conducted semi-structured interviews with patients who participated in a 6-session Zentangle® program accomplished monthly over six months in a Personality Disorders Unit. A total of 15 patients were interviewed for this study. Smith, Flowers & Larkin method was applied for evaluation in the data analysis.

**Results:**

Based on our findings, three categories were extracted: As you sow, so shall you reap (participants reported improvements in concentration, relaxation, interpersonal relationships, and interaction with their environment as well as positive experiences toward acceptance and change); Many hands make light work (patients admitted feeling better in a group and developed group membership. They described how a group environment influences individual behavior); Drawing your own path (this method provides a medium for self-expression and self-knowledge through drawing, improving well-being through emotional expression, enhancing creativity, and increasing self-confidence).

**Conclusions:**

By practicing Zentangle®, patients achieve behavioral responses such as flexibility and adaptability, reaching greater emotional well-being through anxiety management, impulse control, learning to cope with problems, or improving self-esteem or concentration. Mental health nursing plays a critical active role in the comprehensive treatment of BPD, as well as the mobilization and coordination of complementary and diverse interventions.

## What is known?

• Many people around the world practice the Zentangle® Method, which is related to physical and emotional well-being. Very few studies have been done using this method within the mental health field, and it is not currently known what impact practicing the Zentangle® Method can have on patients diagnosed with borderline personality disorder (BPD).

## What is new?

• Study participants described changes starting from their first sessions. Their ability to concentrate improved, they could relax, and they manifested positive feelings toward their group mates and a more significant commitment towards therapy and their environment.
• During the exercises, participants appeared to experience acceptance and a shift in thought about making mistakes. There’s a mindset shift towards negative experiences such as frustration, guilt, or suffering through the active search for alternatives and a reduction in self-imposed expectations.
• This method provides a medium for self-expression and self-knowledge through drawing, improving an individual’s confidence and well-being through emotional expression, the development of creativity, and skill enhancement.

## 1 Introduction

Borderline personality disorder (BPD) is a mental health diagnosis characterized by a general behavioral pattern of impulsivity, emotional instability, and changes both to interpersonal relationships and self-image. Patients diagnosed with BPD often suffer from a severe deterioration in their functioning and serious difficulties in controlling their behavior, which can lead them to react explosively, producing a high risk of self-harming behavior. Furthermore, these patients have a high risk of comorbidity with other psychiatric disorders (anxiety disorders, mood disorders, substance use, eating disorders, high risk of suicide, etc.). All of these situations are related to excessive use of health resources and a high socio-health cost [[1], [2], [3]]. Treating individuals with BPD poses a significant challenge in mental health intervention due to the difficulty of treatment and the considerable complexity in creating an effective therapeutic relationship, implying substantial suffering for the individual. The study shows that treating this complex disorder requires a multidisciplinary and multidimensional approach, in which the general recommendation is to conduct a psychotherapeutic treatment as the first line of treatment [4]. The therapy that stands out the most for its proven effectiveness is Dialectical Behavior Therapy (DBT), developed to specifically treat the clinical presentation of BPD utilizing a combination of cognitive behavioral therapy (CBT) and support strategies, as well as elements of Zen practice and dialectical philosophy [5,6].

Within the multidisciplinary team that treats these patients, mental health nursing, or psychiatric nursing, offers specific competencies and, as such, plays an essential role in the comprehensive treatment of BPD. The mobilization and coordination of various complementary therapies to address patient care that BPD patients require have stemmed from nursing evaluations. These treatments are directed toward health targets such as controlling anxiety, controlling impulses, coping with problems, reducing low self-esteem, improving social interactions, reducing loneliness and the risk of suicide. With the intention of developing new nursing interventions that enable the patient to achieve these objectives, emotional regulation strategies, mindfulness or relaxation techniques appear, highlighting a new tool, Zentangle® [[7], [8], [9], [10], [11]].

The Zentangle® Method is described by its creators Rick Roberts and María Thomas, as “a form of meditative art”. It consists of repeating basic elements following a pattern to easily and mechanically reach a high concentration level similar to that of a meditative state. The different compositions of geometric shapes, curves and repetitive lines, called “tangles”, are drawn within a thread that gives structure to the work. This method is supported by the theory that there are no rough drafts in life and, as such, there are “no mistakes,” only opportunities to make different decisions. The key is in the process itself and the step-by-step approach; the intention is for patients to enjoy the practice without focusing on or thinking of the result [12,13].

To practice Zentangle®, it is just needed white paper in a square format measuring 8.89 cm on each side, a pencil, a pen, and a marker. The drawing is always in black and white. It must be kept in mind that the Zentangle® has to be done in a single session and can’t be deleted during the creation process [[12], [13], [14]].

In its early stages, Zentangle® was compared to Zen meditation. The featured activity encourages individuals to stop and be present during the drawing exercise. It was also later associated with mindfulness. Both techniques promote a state of consciousness focused on the present with no judgments where anything that arises is accepted as it is [14]. They also improve mindfulness skills, such as the ability to act with awareness, concentrate, and reduce distractions without judging experiences, as well as develop the capacity to describe and categorize experiences [[15], [16], [17]].

The results obtained from this method suggest that it can be considered a mindfulness and meditation practice that promotes self-awareness, insight, and problem-solving and can positively impact physical and emotional well-being [18,19]. Some of the additional potential benefits described include reducing anxiety [[20], [21], [22]], improving concentration and fine motor skills [21], as well as a medium for self-care [23,24].

In healthcare field, and more concretely in mental health, research using the Zentangle® Method is limited. Since this is a recently developed method, the research must still examine the effectiveness of this practice and the elements involved in its development. This study seeks to contribute to the insights in the field, with the primary objective of describing the impact of its practice on individuals diagnosed with BPD, describing the emotions it generates, and detecting the possible benefits or challenges during the treatment process as additional specific objectives. This intervention has not been previously studied in this clinical population.

## 2 Methods

### 2.1 Study design

The present study utilizes a qualitative research focus with an interpretative phenomenological approach based on Heidegger’s assumptions, to identify what individuals experience with the practice of the Zentangle® Method and to determine how experiences are perceived. The exploratory qualitative approach is suitable for this topic since it allows one to explore the person’s consciousness, that is, to understand the essence itself, the way of perceiving life through experiences and the meanings that surround them, not only through conversations, but also in what lies behind what is not said.

### 2.2 Setting and participants

**Fig. 1:**
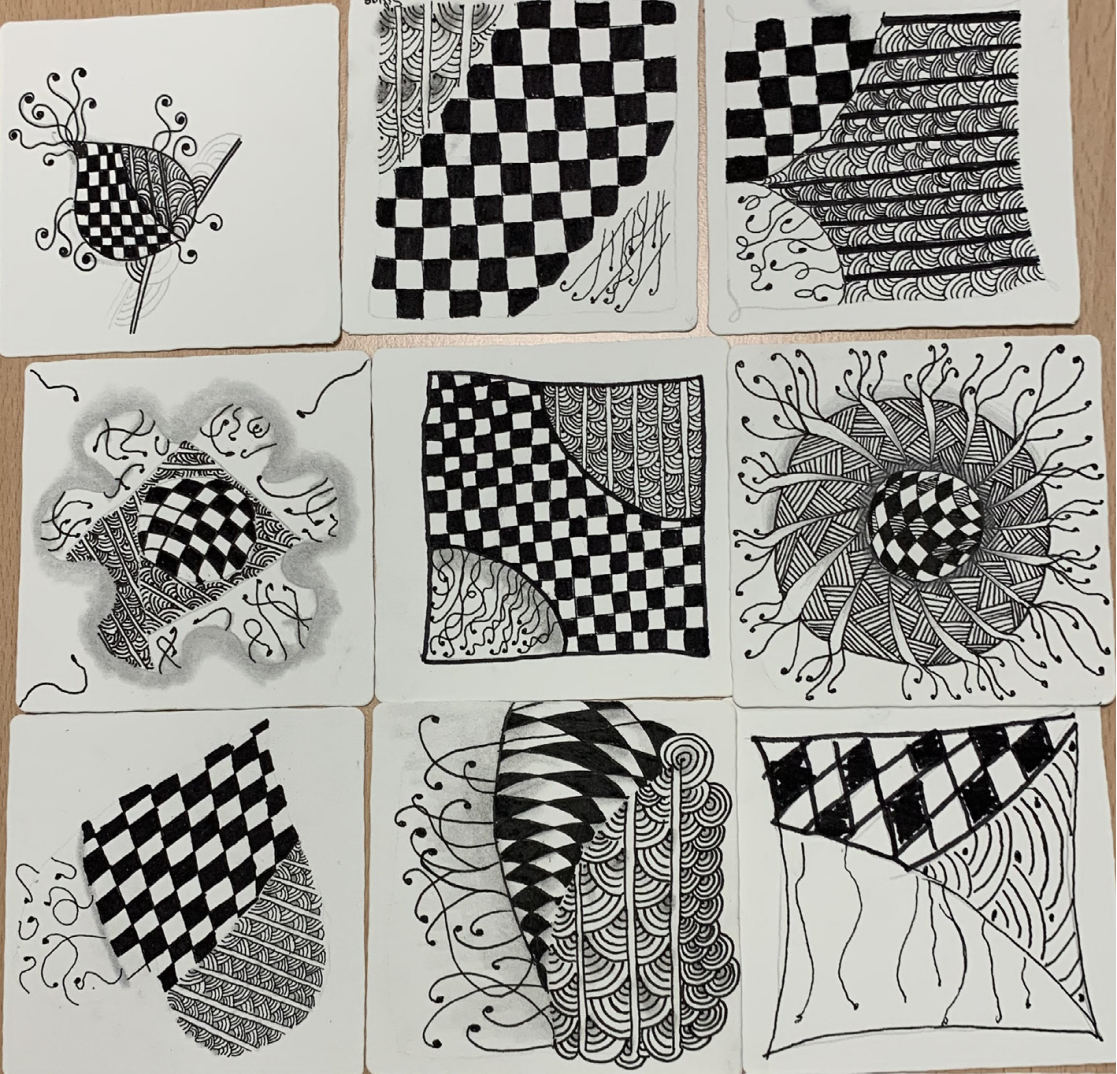
Examples of artwork drawn by patients.

The present study was conducted in the Personality Disorders Unit (Unidad de Trastornos de la Personalidad [UTP]) at Dr. Rodríguez Lafora Hospital in the Community of Madrid (Spain) between January and June 2023. Patients admitted to the unit are diagnosed with BPD and present an instability degree that is not possible to contain on an outpatient basis. These patients are characterized by difficulty in emotional management, impulsivity, emptiness feelings, problems making decisions, self-harm, substance abuse, etc. Admission to the UTP unit consists of an intensive 6-month-long hospital treatment program, which has scheduled sessions that are voluntary and based on the general operating principles of a therapeutic community [1]. Based on the Zentangle® Method, which is detailed in Table 1, the nursing intervention was conducted with a monthly periodicity so that when each patient finalizes treatment, they had participated in a total of 6 sessions. All patients admitted to the unit could participate in the sessions if their psychopathological state allowed them to focus on the activity. The number of participants in the session could be between 3 and 14 people. Each session, lasting 1 h, began with an introduction, explaining the basic rules and the method, detailing the three tangles to be drawn in that session. Afterwards, the Zentangle® was carried out individually, and finally, a reflection would be shared. Examples of artwork drawn by patients can be found in Fig. 1.

**Table 1:** Description of the Zentangle® method nursing intervention.

Introduction (10 min)	Presentation of the activity and a brief introduction to the method Reminders of basic rules: - There are no mistakes - It is abstract - Focus on the present moment and on each line drawn without thinking or looking for a specific result The step-by-step process for the tangles in the session is explained, as well as the shading for each one of the tangles. Reminders of the steps to follow: 1. Draw the border 2. Draw the string 3. Draw the tangles (with a pen) 4. Shade the tangles 5. Sign the front of the drawing 6. Write how you felt on the back of the drawing
Exercise (40 min)	Soothing music is played A brief relaxation exercise is given - Tension is released from the neck and arms - 5 deep breaths are taken - Appreciation of the present moment Initiation to freestyle drawing (The nursing staff is responsible for guiding and answering any possible questions or challenges that may arise, continuously using a motivational and supportive approach. The intervention is aimed at building adaptative coping strategies).
Winding down and reflection (10 min)	Each participant explains how they have felt during the exercise. All the drawings are displayed together in the form of a combined mosaic. 5 min are dedicated to the appreciation, meditation, and gratitude of group work.

The patients did not have any previous contact with the interviewer to clear the way for participants to express themselves as openly as possible. The COnsolidated criteria for REporting Qualitative research (COREQ) checklist was followed throughout this study [25].

Purposeful sampling was the method conducted for the sample selection. Patients diagnosed with BPD with different ages, occupations, sociocultural level and family structure were interviewed using the principle of maximum variation sampling to ensure comprehensiveness and diversity of opinions. As stated in the literature, the prevalence of BPD is higher in women than in men. During the study period, all patients admitted to the unit were women, except for a man who discontinued treatment after two months.

The patients (females, aged 18–65) admitted to the UTP unit, diagnosed with BPD, and participating in the Zentangle® based nursing intervention, were informed verbally and in written form about the study. Patients with language difficulties and those whose psychological state prevented them from communicating their experience were excluded from the study. Also, those who didn’t complete the 5 Zentangle® sessions weren’t eligible for the study. Data saturation was reached within thirteen interviews; this was corroborated by conducting two additional interviews and verified by the principal investigator and the research collaborator. Two of the patients were not interviewed due to scheduling conflicts, which resulted in their withdrawal from the study. Finally, the sample comprised 15 participants, all women, between 19 and 43 years old (average 36.6). They all attended more than five Zentangle® sessions (average 5.66 sessions). Participants identified anxiety and relationship issues as their main difficulties; thirteen of them mentioned low self-esteem, eight admitted isolation and seven of them reported lack of self-care.

### 2.3 Ethical considerations

The study was carried out according to the ethical principles by which all research is guided. The participants were advised both verbally and in writing regarding the study’s objectives and possible benefits and harms due to their participation. Additionally, questions and concerns were addressed, and an informed consent form was provided to ensure voluntary participation and the recording of the interviews. It was outlined that participants could withdraw from the study at any time. All the data collected were anonymized and stored electronically, encrypted, and password protected to preserve the confidentiality of participants.

By the same token, data obtained may only be used and presented as anonymous data in scientific meetings or health congresses, or publications preserving the participants’ privacy at all events. The study obtained the approval of Institutional Review Board of La Paz University Hospital (Approval no. 2X-2XX2).

Being a registered method, the teaching and practice of Zentangle® must be carried out by a Certified Zentangle Teacher (CZT). For the development of the study, there were two CZTs. In addition, two of the study’s researchers have subsequently been certified.

### 2.4 Data collection

Data collection was conducted through semi-structured in-depth interviews of 30–45 min of duration. The principal investigator, a female nurse, specializing in mental health with specific training in the Zentangle® Method, was responsible for conducting the interviews. A second person, who specialized in mental health and served the role of observer, transcribed the information. All interviews were conducted in an office in the UTP unit the day after finishing the last Zentangle® session and complied with the appropriate conditions for the study. The interviews took place during the patients’ free time not to interfere with their dynamic treatment regimens and were recorded with the consent of the interviewees.

The interviews were conducted using an interview script based on the objectives of this study and other qualitative studies reviewed by all authors. The interviewer sought to allow participants to describe their experiences freely and in the greatest detail possible, using open-ended questions complemented with additional questions to seek more detail or clarification. Question example: What changes have you noticed in yourself and Zentangle® practice after the sessions? How would you describe the relationship felt with other participants? What environmental factors do you consider may influence you during practice? How does Zentangle® practice affect your mood? How do you feel practicing Zentangle®? When do you practice Zentangle®?

Transcripts were returned to participants so that they could verify that their testimony was as they had expressed.

### 2.5 Data analysis

A subject matter analysis of the data gathered through interviews was conducted to analyze the data. Following the guidelines outlined by Smith et al. [26], a complete transcription, revision, and first and second reading of the interviews were completed for posterior coding. The researchers repeatedly read the transcribed texts, immersed themselves in the data, and became familiar with it, gaining a comprehensive understanding of patients’ experiences practicing Zentangle®. Passages or relevant and vital texts were extracted and organized into categories and subcategories. The different categories were analyzed thoroughly, and the data was identified, classified, and sorted to facilitate the study and its analysis. Finally, the full texts were combined, corroborating the conclusions drawn from the study and allowing for a better overall understanding of the discourse. A second analysis was conducted in parallel by the research collaborators to ensure rigor and research quality, through which similar conclusions were drawn [26,27]. All the researchers participated in defining and naming the topics. The reflections of researchers were embedded throughout the whole study process.

Results were presented to participants so that they could mention if they felt identified and add any comments.

### 2.6 Trustworthiness

To ensure the study’s trustworthiness, several measures were implemented: first, the interviewer and second researcher are Zentangle® certified teachers and mental health nurses who could establish good relationships with patients. Second, the interviews with patients were conducted in a spare room during the patient’s free time in order to prevent interference during the conversations. Third, one of the researchers has experience in qualitative research, having attended several courses and published various qualitative papers. The final topics and subtopics were obtained by triangulation between the three researchers. In addition, results were presented to patients to confirm that their experience was reflected. Finally, quotes from different interviewees were reported to ensure the trustworthiness of the findings.

## 3 Results

The results obtained from the analysis of the patients’ experiences, collected through interviews, have given rise to three themes and several subthemes, which are described below in detail. The main verbatims of the experiences and examples of artwork drawn by patients are gathered in Table 2.

**Table 2:** Literal quotations from interviews by category.

Topics	Subtopics	Participant Testimonials (Verbatims)

As you sow, shall you reap	Here and now	*“I’m a very nervous person, it’s challenging for me to concentrate, and my brain doesn*’*t stop …, and with Zentangle® I can concentrate on drawing. I focus on what I have to do for each step, and I don*’*t think of anything else, and I can abstract myself. I don’t know if10 minhave passed or 25 …”* (Participant 11) *“I don’t like not finishing on time; it frustrates me, but it’s good that there’s a time limit … It has helped me to keep getting better.”* (Participant 15)
	Pathway to calm	*“This is the only one [relaxation technique] that relaxes me and allows me to forget what’s on my mind. I just don’t think of anything; I’m deep into the exercise, really aware … I notice that I leave the session much calmer.”* (Participant 14) *“The thing with me is that relaxation exercises are always dreadful for me … relaxing in itself, which is so difficult for me, is impossible … relaxing in of itself doesn’t relax me, I’m nervous just doing it … but it’s like nerves from curiosity and wanting to know what the session will result in today.”* (Participant 13)
	Imperfect perfection	*“At the beginning, it annoyed me, now it calms me down … I allow myself to make mistakes and continue.”* (Participant 2) *“Realizing that there are no rules or demands, it doesn’t have to be nice or perfect … it didn’t turn out well, and it didn’t matter … that’s where I noticed I felt almost relieved … So, I think it’s good that you can’t erase it; it helps you accept whatever turns out.”* (Participant 15) *“We should apply this to real life. For example, the feeling of guilt that many of us here carry is very palpable. We can make poor decisions … sometimes you can change them, and sometimes you can’t. But you have no choice but to move on.”* (Participant 10)
Many hands make light work	Best in group	*“It surprises me, and I like it (Zentangle® practice at hospital); I’ve bought myself a drawing block and pens. I put music on at home, but I don’t manage to get it done (home alone). I think it*’*s the environment, the company, the fact that everything is set up so well; it’s like creating the moment and sharing it.”* (Participant 3) *“I’ve tried several times at home, and I do like it, but it’s not the same. In the hospital, being with my colleagues and the guidance they give us, makes me enjoy it more than alone.*” (Participant 12)
	Connecting people	*“I like seeing the final showing; it’s something we’ve all created together. Seeing how different each of our drawings are, each detail, how each of us interprets things, and that despite the differences, how we complement one another as a whole.”* (Participant 2) *“At the beginning, I didn’t like it when we’ve display everyone*’*s work together because of my own drawing, it was horrible in my eyes. But now I like seeing what they’ve done … There’s a lot of camaraderie; we always say nice things to one another.”* (Participant 4)
	Growing up together	*“I think that it’s one of the few activities where we respect each other … so I feel comfortable.”* (Participant 9) *“In this exercise (#2), I complained, and MC got mad at me … Now, I’d get as mad as MC if someone bothered me. I started to understand it over time … that respect for the person sitting next to you, that in reality what you also want is that others respect you too.”* (Participant 6)
Drawing your own path	Express yourself	*“The drawings reflect your personality, and you can also tell how each person is doing. One day I arrived super angry, and when I was done, I’d released it all. It allowed me to blow off steam … Also, every time we do this, I, at least, start realizing certain things.”* (Participant 5)
	Step by step	*“The rules are good because then you don’t draw a blank, but everyone draws the tangle in their own way. The proof is that, in the end, each tile is completely different. I didn’t consider myself a creative person, but I’ve been getting better each time I get more daring and try different things.*” (Participant 6)
	Rome wasn't built in a day	*“I*’*ve always been bad at drawing … And it makes it easier for me to start with a designated space, you’re given a tangle to draw.”* (Participant 1) *“The shading is what is difficult for me. I*’*m still getting the hang of it. The fact that it’s slowly starting to turn out … makes me feel good.”* (Participant 10)
	Beauty is in the eye of the beholder	*“There’s a before and after as you being to get the hang of it and you start to improve.”* (Participant 13) *“In the first drawing I made, I got very frustrated. In fact, I didn’t even want to show my work … It’s something I’ve gotten better at.”* (Participant 10)

### 3.1 As you sow, so shall you reap

#### 3.1.1 Here and now

All participants agree that they feel focused and pay attention to the task and how to execute it during the Zentangle® exercises. In most cases, this makes it easier for them to let go of other thoughts. Patients describe how sometimes they get distracted during the exercise but can quickly refocus on their drawing. Seven of the patients identified changes in their level of concentration with each session, indicating that the sessions became easier as they became more experienced, albeit their state prior to the session could influence the level of concentration reached during the exercise.

For the patients, this state of concentration generates a feeling of losing control over the elapsed time. In general, Zentangle® practice is seen as a positive exercise except for three of the participants who agree that when there are 5 min left to finish the session, not having had a true sense of the elapsed time could become an inconvenience, bringing on anxiety or pressure to finish and even frustration for not finishing on time. With the passing of each session, patients begin to accept the circumstances and begin to see the time limit as necessary.

#### 3.1.2 Pathway to calm

The participants describe themselves as restless individuals with high anxiety levels and severe difficulties in reaching a state of relaxation. Despite their self-reported difficulties, half of the participants did experience a state of relaxation, connecting it to their concentration level, allowing them to “stop their mind.”

Among the participants who did not reach a state of relaxation, they described a state of “positive” anxiety combined with excitement and curiosity for the final result or feelings of anxiety concerning their baseline state of mind. All the participants compared this meditative method with other relaxation techniques, expressing a better tolerance by having to focus on specific elements.

Several participants cite other factors that could affect the degree of relaxation the exercise provides, such as the tangles selected for each session, the background music, or the available workspace.

#### 3.1.3 Imperfect perfection

The participants, who speak in depth about the meaning of the basic rule of “there are no mistakes,” agree in their accounts that during the first sessions, this rule comes across as a constraint that could generate feelings of anger and frustration because their perception is that they do make mistakes. Throughout the sessions (#3 and 4), the participants explained that they continued to make mistakes, but their perception of it changed, moving past frustration to relaxation and relief. They speak of acceptance as a mechanism for change, taking pressure off themselves about the final result. The participants that delve deeper into this point also speak about being able to extrapolate their learnings and the acceptance of experience acquired using the Zentangle® Method to their daily lives, allowing themselves to lower their expectations, make mistakes, and let go of self-punishment were also ways of reducing guilt and suffering.

Another mechanism that generates a change from negative to positive emotions when making mistakes is the understanding that there is an opportunity to make different decisions. Six participants adopted this approach, generating curiosity within themselves and building confidence when facing alternatives and decision-making, creating a space where trial and error can occur safely.

### 3.2 Many hands make light work

#### 3.2.1 Best in a group

The participants describe differences between group work and individual work in practice. All the participants agree on the positive effect of the structure, the ceremony, and the company to “create the moment,” which they also link to being beneficial on all levels.

#### 3.2.2 Connecting people

Participants describe a calm environment where there is mutual respect and where they are comfortable and feel a sense of connection and understanding, camaraderie, and support during the session.

At the end of the study, fourteen participants shared these sentiments and felt curiosity about group work. They describe the combined mosaic at the end of the sessions as a connecting element for the group. Four participants described a change in opinion on this aspect of the sessions. In the first sessions, they were embarrassed and uncomfortable when showing their work to the group. These feelings have evolved to attributing greater importance to group work and focusing less on individual work.

One participant felt negative about group work despite having several sessions, experiencing envy or a tendency to compare herself to others. These feelings were accepted as part of the process, not judged, and seen as an opportunity for personal work.

#### 3.2.3 Growing up together

Participants often compare their behavior during other therapy sessions, indicating greater respect for the rules and fellow participants during the Zentangle® sessions. One of the participants describes a change in her behavior during the therapy sessions, indicating that she has a greater understanding of the importance of remaining silent and respecting others’ time after completing several sessions. The most striking element of the group influence is that working in a calm environment lets the participants focus more on their work.

Further, combining the mosaic of the completed drawings at the session’s end and receiving support and positive feedback from groupmates can influence an individual’s self-esteem and how they feel about their work.

### 3.3 Drawing your own path

#### 3.3.1 Express yourself

All participants speak of the Zentangle® Method as a means of expression. They describe it as a rewarding activity in which an individual's emotional state or personality is revealed without intending to do so. This lack of intention to express their emotions provides another perspective in which they can observe aspects of themselves that were unknown to them. Participants spoke of this self-knowledge as a process gained through practice and the importance of their sense of well-being achieved through emotional expression.

#### 3.3.2 Step by step

Participants indicate that their creativity is further developed with each passing session, particularly once they get a better handle on the technique and understand that the instructions are guidelines that can be interpreted in multiple ways. They allow themselves to adopt changes and alternatives more confidently, even among individuals who did not consider themselves creative previously.

#### 3.3.3 Rome wasn’t built in a day

Eleven participants had no experience with drawing and explained that this method is an easy way to start as it allows them to do something they always thought they “did poorly” and avoid mental block when they did not know what to do. Despite expressing ease, eight participants described having difficulty in the first therapy sessions, including handling materials or completing specific tangles. These difficulties are generally resolved through practice. One participant mentioned developing creative skills and linked it to a greater focus on well-being.

Four participants appear to have had drawing skills before the intervention. They describe Zentangle® as an easy way to start drawing again, improve their creativity, or add finishing touches to their work.

#### 3.3.4 Beauty is in the eye of the beholder

Starting from the beginning, most participants describe the sessions as “something beautiful,” generating feelings of joy, well-being, and personal pride, which could positively influence self-esteem. Participants also describe a positive evolution in self-esteem with each passing session. The final result can have a significant influence on participants.

Five participants were not always satisfied with the final results, sometimes experiencing anger, frustration, or envy, which were most prominent in the first therapy sessions.

## 4 Discussion

If the objectives proposed at the beginning of the research study are considered, some answers come up regarding the experience of practicing the Zentangle® Method for patients with BPD. A thematic analysis of reported experiences broadens prior knowledge of the of the Zentangle® Method practice. This analysis determined there could be various foci for departure to develop further vital aspects of treating patients diagnosed with BPD.

All participants agree that they find themselves focused on the exercise, paying attention to what must be done and how to do it, making it easy to set aside other thoughts, in particular, intrusive and persistent thoughts that are negative and exhausting for patients with BPD [1]. Consequently, participants indicate a greater tolerance to the Zentangle® Method than other relaxation techniques. This greater tolerance brings excellent value at a therapeutic level as it aids in reducing anxiety in individuals who have a greater aversion to more conventional relaxation techniques.

Despite their self-reported difficulties in reaching a state of relaxation, some participants reached this state through an initial state of concentration that allowed them to “stop their mind.” This improvement in anxiety management, as well as affective well-being, was also found in Stojcevski et al.’s study [28].

The Zentangle® tangles could contribute to a patient’s ability to change their focus, similar to the concentration process in Zen meditation. The abovementioned improvements in physical and mental well-being occur when an individual shifts their attention and awareness from symptoms (both psychological and emotional) and focus on an alternate activity [29], similar to what study participants experienced primarily on a psychological level, also mentioned by Chan et al. in their research [30].

Regarding the basic rule of “there are no mistakes,” some participants shift from feeling angry and frustrated about making mistakes to executing the activity in a relaxed and relieved state.

The Zentangle® Method does not require art to have any meaning; it is a medium for abstract expression that, in turn, removes the anxiety surrounding creating representational art [19]. On the one hand, the participants link this shift to a reduction in self-imposed expectations, taking pressure off themselves regarding the result. On the other hand, participants connect this to acceptance and perceive the “there are no mistakes” guideline as a normal part of the whole process without having any negative emotional responses. Participants report that being able to extrapolate their learnings and the acceptance of experience to their daily lives is a benefit as it reduces self-imposed demands and allows space for making and accepting mistakes without punishing themselves, which then lessens any feelings of guilt or suffering usually felt daily [1]. The phenomenon corresponds with various publications discussing the practice of mindfulness within the Zentangle® Method. Several studies attest that mindfulness supports psychotherapy treatment in patients with BPD, and the studies correlate this practice to clinical improvement and decreased emotional reactivity [17,31,32]. In addition to accepting mistakes, they can be viewed as an opportunity to make different decisions. This process of making different decisions during the sessions helps participants balance acceptance and change, a vital aspect of the clinical approach in nursing for patients with BPD [15,16,33].

Another thematic cluster that stands out in the participants’ discourse is their group experience, which was positive overall as it created a comfortable and safe space of mutual respect. These perceptions create an environment where participants can be calm and feel safe, another critical aspect of creative and group therapy settings [34]. Feelings of mutual understanding and connection, appreciation, camaraderie, and mutual support appear during the group therapy sessions. Building and maintaining social connections is particularly difficult for patients with BPD [1,2]; hence the importance of experiencing positive feelings, such as a sense of belonging and group membership when considering interpersonal relationships. Chen et al. trial showed significant improvement in social interactions in patients with schizophrenia who practiced Zentangle® as well [35].

All participants in this study describe the practice of the Zentangle® Method as a medium of expression and a path to self-knowledge in which their state of being or personality is revealed without the intention of doing so. Zentangle® practice, in turn, creates an opportunity for individuals to express themselves in unique and personal ways, highlighting the importance of emotional expression and the feeling of well-being it generates. In 2016, Kopeschny already connected this method with Art Therapy, the benefits of which are outlined in treatment for BPD as the reduction of psychopathology and the development of coping skills [19,36].

Participants describe the Zentangle® Method as an easy way to start drawing, which prevents mental blocks through its established framework and allows creativity to flow. They sometimes feel more confident once they begin to master the technique. The repetition required leads to developing self-confidence, mastery, and proficiency, where trial and error can be applied confidently. Most participants describe the process as a rewarding experience in which the final result and the growth experienced generate feelings of joy, well-being, and personal pride, which positively influence self-esteem. As early as the first few sessions, some patients can perceive some benefits of practicing the Zentangle® Method, describing growth and overall improvement with each passing session. It should be noted that in half the cases, this growth would be necessary to discern any benefits, which could pose an issue for patients with BPD and their treatment due to their strong tendency to abandon activities or irregular attendance, which are related to the characteristic impulsiveness of these patients [37]. Furthermore, during the first sessions, most difficulties and negative perceptions may arise, but patients gain experience with their regular attendance. Some difficulties include handling materials, understanding the rules, not finishing on time, making mistakes, or negatively perceiving the final result. These difficulties could cause frustration, anger, shame, or discomfort. The key element of regular attendance is vital, given that participants state that these challenges and negative feelings decrease or are even overcome with each passing session. This key element could be attributed to improved ability in handling materials and mastering techniques, developing creativity and a greater sense of confidence, or the process of acceptance of experience, or thanks to the support and feedback received in group therapy.

This research study is an initial attempt to understand how patients with BPD experience the practice of the Zentangle® Method and its implications for nursing practice. These findings, while of an exploratory nature, contribute to the current understanding of this method and enable a new field of work for mental health nurses, promoting autonomous interventions and expanding their scope of action. Zentangle® can be a new learning method for complex patients, such as those with BPD, who can use it as a self-regulation strategy.

*Limitations.* The Zentangle® practice nursing intervention is included within a 6-month-long comprehensive treatment program for BPD patients, comprising multiple complementary therapies and activities that aim to improve a patient’s condition. As such, while the interviews focused on elements of the Zentangle® Method, extracting this intervention’s effects in isolation is problematic. Furthermore, all the participants were women, who were not randomly selected; as such, the results could be biased due to their voluntary participation and gender roles. No articles comparing Zentangle® practice with another relaxation method were found.

## 5 Conclusions

The current study provides a comprehensive perspective of the lived experience of Zentangle® practice in patients with BPD and the possible beneficial or detrimental factors that can influence their treatment. Although there is still a long road ahead, this project provides a greater understanding of the subject and generates new ideas for clinical applications. Mental health nursing, which requires specific expertise and abilities for the treatment of patients with these characteristics, plays a critical active role in the comprehensive treatment of BPD, as well as the mobilization and coordination of complementary and diverse interventions, such as Zentangle® practice, to achieve greater behavioral flexibility, which, in turn, can provide a greater sense of emotional well-being to an individual. Greater well-being may also be achieved through anxiety management, impulse control, learning to cope with problems, or improving self-esteem or concentration.

The present study can only suggest new clinical applications and hypotheses for testing in future research due to the subjective nature of qualitative findings, based in this case on the participants’ interpretations of their experience and which are then, in turn, interpreted by the principal investigator and the research collaborator.

## 6 Recommendations

In future research, it would be of interest to conduct a qualitative analysis on a more significant scale to include both men and women in order to get a gender perspective. Also, it would be necessary to delve deeper into the long-term effects of continued Zentangle® practice and to expand on factors and mechanisms that lead to change in patients. It is recommended that the role that nurses play within the intervention and their influence on patients be explored further. Lastly, future research could also include quantitative tracking of the perceived benefits of Zentangle® practice.

## CRediT authorship contribution statement

**Ana Morales-Alonso:** Conceptualization, Methodology, Validation, Formal analysis, Investigation, Data curation, Writing - original draft, Writing - review & editing, Project administration. **Ángela Iglesias-de-la-Iglesia:** Conceptualization, Formal analysis, Data curation, Writing - review & editing. **Miriam Alonso-Maza:** Conceptualization, Methodology, Validation, Formal analysis, Data curation, Writing - review & editing.

## Funding

Nothing to declare.

## Data availability statement

The data generated and scrutinized throughout this study have been integrated into this published article.

## Declaration of competing interest

The authors have declared no conflict of interest or any potential competing interest.
