# Supplementary material for: A nursing intervention based on the Zentangle® method: Experiences of patients diagnosed with borderline personality disorder
Source: Int J Nurs Sci. 2024 Mar 11;11(2):205–13. doi: 10.1016/j.ijnss.2024.03.004 (PMC11064625; doi:10.1016/j.ijnss.2024.03.004)
Supplement: Multimedia component 1 [file mmc1.docx]

基于Zentangle® 艺术治疗方法的护理干预：边缘型人格障碍患者的体验

Ana Morales-Alonso, Ángela Iglesias-de-la-Iglesia, Miriam Alonso-Maza

【摘要】

**目的** Zentangle®艺术治疗方法被应用于心理健康相关领域，尚未在边缘型人格障碍患者中得到验证。该研究旨在分析边缘型人格障碍患者接受Zentangle® 艺术治疗方法后的练习体验。

**方法** 采用现象学解释方法，对患者进行半结构化访谈。患者均已在西班牙某医院的人格障碍治疗室接受了每个月1次、共6次的Zentangle® 艺术治疗。该研究共访谈了 15 例患者。Smith、Flowers和 Larkin方法用于资料分析中的评估。

**结果** 根据研究结果，共提炼出3个类别的主题：有耕耘即有收获（参与者报告了注意力、放松、人际关系、与环境互动方面的改善，以及接受和改变的积极体验）；众擎易举（患者认可在团体中感觉更好，并建立了相互联系；他们也描述了团体环境对个人行为的影响）；画出自己的轨迹（通过绘画方式提供了自我表达和自我认识的媒介，通过情感表达提升了幸福感，提高了创造力，增强了自信心）。

**结论** 通过练习 Zentangle®，患者可以作出灵活性和适应性等行为反应，通过焦虑管理、冲动控制改善情绪健康，学习应对问题，提高其自尊或注意力。心理健康护理在边缘型人格障碍患者的综合治疗及协同多样化补充性干预措施中发挥着积极作用。

【关键词】艺术治疗； 边缘型人格障碍； 心理健康； 护理； Zentangle

通信作者：Miriam Alonso-Maza， E-mail: [miriamalonsomaza@gmail.com](mailto:miriamalonsomaza@gmail.com)
